# Amyloid-β PET—Correlation with cerebrospinal fluid biomarkers and prediction of Alzheimer´s disease diagnosis in a memory clinic

**DOI:** 10.1371/journal.pone.0221365

**Published:** 2019-08-20

**Authors:** Ebba Gløersen Müller, Trine Holt Edwin, Caroline Stokke, Sigrid Stensby Navelsaker, Almira Babovic, Nenad Bogdanovic, Anne Brita Knapskog, Mona Elisabeth Revheim

**Affiliations:** 1 Department of Nuclear Medicine, Oslo University Hospital, Oslo, Norway; 2 Institute of Clinical Medicine, University of Oslo, Oslo, Norway; 3 Department of Geriatric Medicine, The Memory Clinic, Oslo University Hospital, Oslo, Norway; 4 Department of Diagnostic Physics, Oslo University Hospital, Oslo, Norway; 5 Department of Life Science and Health, Oslo Metropolitan University, Oslo, Norway; 6 Department for Neurobiology, Caring Science and Society, Division of Clinical Geriatrics, Karolinska Institutet and Karolinska University Hospital, Stockholm, Sweden; Nathan S Kline Institute, UNITED STATES

## Abstract

**Background:**

Alzheimer’s disease (AD) remains a clinical diagnosis but biomarkers from cerebrospinal fluid (CSF) and more lately amyloid imaging with positron emission tomography (PET), are important to support a diagnosis of AD.

**Objective:**

To compare amyloid-β (Aβ) PET imaging with biomarkers in CSF and evaluate the prediction of Aβ PET on diagnosis in a memory clinic setting.

**Methods:**

We included 64 patients who had lumbar puncture and Aβ PET with ^18^F-Flutemetamol performed within 190 days. PET was binary classified (Flut+ or Flut-) and logistic regression analyses for correlation to each CSF biomarker; Aβ 42 (Aβ_42_), total tau (T-tau) and phosphorylated tau (P-tau), were performed. Cut-off values were assessed by receiver operating characteristic (ROC) curves. Logistic regression was performed for prediction of clinical AD diagnosis. We assessed the interrater agreement of PET classification as well as for diagnoses, which were made both with and without knowledge of PET results.

**Results:**

Thirty-two of the 34 patients (94%) in the Flut+ group and nine of the 30 patients (30%) in the Flut- group had a clinical AD diagnosis. There were significant differences in all CSF biomarkers in the Flut+ and Flut- groups. Aβ_42_ showed the highest correlation with ^18^F-Flutemetamol PET with a cut-off value of 706.5 pg/mL, corresponding to sensitivity of 88% and specificity of 87%. ^18^F-Flutemetamol PET was the best predictor of a clinical AD diagnosis. We found a very high interrater agreement for both PET classification and diagnosis.

**Conclusions:**

The present study showed an excellent correlation of Aβ_42_ in CSF and ^18^F-Flutemetamol PET and the presented cut-off value for Aβ_42_ yields high sensitivity and specificity for ^18^F-Flutemetamol PET. ^18^F-Flutemetamol PET was the best predictor of clinical AD diagnosis.

## Introduction

Alzheimer´s disease (AD) is a progressive degenerative disease of the brain that mainly affect older people. It is the most common form of dementia and as the population grows older the prevalence is increasing. [1, 2]. There is increasing evidence that the typical neuropathological changes in AD start to develop decades prior to onset of symptoms [3–5], hence the recognition of AD as a continuum [6]. These changes are considered hallmarks of AD, namely neurofibrillary tangles and neuritic plaques. Biomarkers for AD reflect these hallmarks and were included into the diagnostic criteria in 2011, although only for research purposes [7, 8]. The recently published research framework recognizes the increasing position of biomarkers in AD and proposes a focus on biologically defining the etiology of AD through the use of biomarkers, rather than defining diagnosis by clinical symptoms [6]. Biomarkers of AD may be measured in cerebrospinal fluid (CSF) or evaluated with positron emission tomography (PET). PET utilizes radioactively labelled amyloid β (Aβ) tracers, or the more recently investigated tau-tracers, which bind to fibrillary forms of Aβ and tau in the brain, respectively. Evidence shows that decreased levels of Aβ consisting of 42 amino acids (Aβ_42_) in CSF or a positive Aβ PET is closely related to Aβ deposition in neuritic plaques [9, 10] while increased levels of phosphorylated tau protein (P-tau) in CSF or a positive tau-PET is closely related to fibrillar tau accumulation in neurofibrillary tangles [11–13]. Elevated total tau (T-tau) in CSF, atrophy on magnetic resonance imaging (MRI) or hypometabolism on 2-deoxy-2-(^18^F)-fluoro-D-glucose (FDG) PET are biomarkers of neurodegeneration which may co-occur with the other hallmarks [6]. Evaluation of biomarkers with PET or CSF may be used to gain confidence in a clinical AD diagnosis [6–8]. ^18^F-Flutemetamol is an Aβ PET-tracer that binds to fibrillar Aβ and it has a similar structure to the widely investigated ^11^C-Pittsburg compound B (PiB) as both are derivatives of Thioflavin-T, labelled with different radioisotopes. Previous studies have shown a good correlation between ^18^F-Flutemetamol binding and neuropathological evidence of Aβ neuritic plaques [14–17], as well as a good correlation between ^18^F-Flutemetamol PET and CSF measurements of Aβ_42_ [18–20]. Consequently, Aβ PET has been used to suggest cut-off values for CSF biomarkers for AD [18, 20]. However, the cut-off values vary as different methods for CSF analysis and different Aβ PET-tracers are used.

The aim of this study was to correlate ^18^F-Flutemetamol PET with CSF biomarkers to assess the optimal cut-off values, to evaluate the effect of the newly implemented cut-off value for Aβ_42_ and to investigate the prediction of ^18^F-Flutemetamol PET on a clinical AD diagnosis.

## Materials and methods

### Study population

This was an observational cross-sectional study consisting of 64 patients who had undergone both ^18^F-Flutemetamol PET and lumbar puncture for CSF sampling as a part of clinical routine in the workup of cognitive complaints between February 2015 and October 2018. Patients with less than 190 days between the two biomarker examinations were included from the memory clinic at Oslo University hospital (OUH), Ullevål. All included patients had signed a written consent form for inclusion in the Norwegian Register of Persons assessed for Cognitive symptoms (NorCog). At the time of inclusion in NorCog, all patients were deemed to have sufficient cognitive capacity to consent. Clinical data was extradited from the NorCog registry. In cases of inadequate information in the registry, the patients’ medical record was consulted. The study, as well as the consent procedures, were approved by the regional Ethics Committee for medical research in the South-East of Norway (REK 2017/1929) and the Data Protector Officer at our institution.

### ^18^F-Flutemetamol PET CT acquisition

All patients were examined using the same PET scanner, Siemens Biograph40 mCT (Siemens Healthineers, Erlangen, Germany). Image acquisition started 90 minutes (range 75–117 minutes) after patients received a bolus injection of 185 MBq ^18^F-Flutemetamol. First a low-dose CT scan was performed for attenuation correction and anatomic information for the PET images. The low-dose CT was performed without contrast enhancement (120 kV, 70 mAs and with slice thickness of 3 mm). PET data were acquired for 20 minutes (four frames of 5 minutes each), except for in one patient where the acquisition time was prolonged to 30 minutes to compensate for a lower amount of activity given (94 MBq). 3D dynamic emission data were reconstructed using a resolution recovery algorithm with time of flight (TrueX with two iterations, 21 subsets and a Gaussian filter with FWHM of 2 mm and a matrix size of 400 x 400). The slice thickness of the reconstructed image series was 2 mm, giving a voxel size of 2 x 2 x 2 mm^3^.

### Qualitative classification of ^18^F-Flutemetamol PET

^18^F-Flutemetamol PET images were qualitatively classified using a Siemens SyngoVia workstation (version VB20, Erlangen, Germany). Images were classified by visual assessment as positive if one of the following 5 regions, in either hemisphere, showed increased cortical uptake; frontal lobe, posterior cingulate and precuneus combined, lateral parietal lobe, lateral temporal lobe or striatum. This was performed according to the validated image reader program [21]. An experienced nuclear medicine physician who was blinded to the clinical information classified PET as positive (Flut+) or negative (Flut-). The study-specific classification was compared to the clinical classification from the patients’ medical record which was performed by two nuclear medicine physicians (one resident and one consultant or two consultants). If there was disagreement between the study-specific classification and clinical classification the images were evaluated by a third nuclear medicine specialist where agreement of two parties led to a final conclusion. All physicians evaluating ^18^F-Flutemetamol PET had completed the validated online electronic training program course supplied by the vendor [21].

### CSF sampling and analysis

A lumbar puncture with measurement of the CSF core biomarkers Aβ_42_, T-tau and P-tau using the ELISA technique with the Innotest kit (Innogenetics, Ghent, Belgium) was performed for all patients. The analysis was done at the laboratory at Akershus University Hospital, Norway. The laboratory is a part of the Alzheimer´s Association QC program for CSF biomarkers [22]. The recommended cut-off value for a normal test was until June 2017 >550 pg/mL for Aβ_42_. As of October 2018, when these data were collected, the recommended cut-off values for a normal test from the laboratory were Aβ_42_ >700 pg/mL [23], P-tau <80 pg/mL and T-tau <300 pg/mL for patients below 50 years, < 450 pg/mL for patients aged 50 to 69 years, and <500 pg/mL for patients older than 70 years. These cut-off values were used to support the clinical diagnoses that were made (see section below).

### Clinical diagnosis

All patients were assessed according to a standardized and comprehensive research protocol including detailed information from the patients and the caregivers about symptoms, previous disorders, use of medication and demographic information [24]. The cognitive function was assessed by several cognitive tests, including the Mini-Mental State Examination (MMSE) [25], the Consortium to Establish a Registry of Alzheimer’s Disease (CERAD) 10-item word list and constructional praxis exercise [26], the Clock Drawing Test (CDT) [27], the Trail Making Tests A and B (TMT A and B) [28], the animal-naming test, the Controlled Oral Word Association Test (COWAT-FAS test) [29–31] and the 15-word short of the Boston Naming Test (BNT) [32]. For the purpose of this study the severity of the cognitive impairment was scored by an experienced rater using the Clinical Dementia Rating scale (CDR) [33]. In addition, the patients underwent a physical examination with blood sample tests and in most cases a magnetic resonance imaging of the brain (MRI) (n = 58).

Diagnosis and staging of cognitive impairment were made by two experienced memory clinic physicians. Diagnoses were made retrospectively based on all available information in medical records, in time-proximity to the PET examination. One of the physicians was blinded for the results of ^18^F-Flutemetamol PET. A third experienced physician was consulted in equivocal cases. All patients were assessed for clinical etiology (AD or non-AD), hereafter referred to as clinical diagnosis, and stage (subjective cognitive decline (SCD), mild cognitive impairment (MCI) or dementia). SCD was diagnosed using the criteria from the Subjective Cognitive Decline Initiative [34]. MCI or dementia, as well as clinical diagnoses, were based on the National Institute of Aging and the Alzheimer’s Association (NIA-AA)-criteria [7, 8]. All patients with probable and possible AD (including mixed AD) according to the NIA-AA criteria were categorized as having clinical AD diagnosis. Patients were thus categorized into the following groups: MCI-AD, MCI-non-AD, dementia-AD, dementia-non-AD and SCD. The etiology for SCD was considered non-AD (SCD-non-AD). These five groups were used for the interrater agreement analysis, while the clinical diagnosis (AD or non-AD) was used for all other analyses.

### Statistical analysis

The statistics were performed using IBM SPSS, version 25. Group differences were explored using t-test if the data was normally distributed, Mann-Whitney test if data were not normally distributed and Chi-square test if there were two categorical variables to be analyzed. Univariate binary logistic regression analyses were performed to assess the correlation of Aβ_42_, T-tau and P-tau in CSF, as well as age and sex, with ^18^F-Flutemetamol PET. The same variables were included as independent variables in a multivariate logistic regression analysis with ^18^F-Flutemetamol PET as dependent variable. We created receiver operating characteristic (ROC) curves of ^18^F-Flutemetamol PET against Aβ_42_, T-tau and P-tau in CSF as well as the ratios of T-tau/Aβ_42_ and P-tau/Aβ_42_ for comparing the performance of the CSF biomarkers and ratios with ^18^F-Flutemetamol PET and to evaluate the optimal cut-off values. Youden´s indexes were calculated to find the optimal thresholds.

Univariate binary logistic regression analyses were performed with ^18^F-Flutemetamol PET, Aβ_42_, age, sex and CDR as independent variables and clinical diagnosis as dependent variable. These independent variables were also included in a multivariate logistic regression analysis to assess their prediction on clinical diagnosis. Multiple models with different combinations of independent variables were tested. The odds ratios from the regression analyses account for a 1 unit increase for each variable. Furthermore, with an increase of x units in the independent variables, the odds ratio is given by Odds ratio^x^. Cohen κ analysis was used to assess the interrater agreement of the qualitative classification of ^18^F-Flutemetamol PET between the study-specific and clinical classifications. Cohen κ analysis was also used for assessing the interrater agreement of the combined stage and clinical diagnosis categories with and without knowledge of ^18^F-Flutemetamol PET classification.

## Results

Patient characteristics, CSF biomarker levels, distribution of diagnoses and cognitive measures for the patient population are listed in Table 1.

**Table 1:** Patient characteristics.

Patient characteristics (n = 64)	
**Sex and age**	
Females, n (%)	32 (50)
Males, n (%)	32 (50)
Age (y), mean (SD, min-max)	66.3 (7.6, 47–77)
Age females (y), mean (SD)	66.2 (7.5)
Age males (y), mean (SD)	66.4 (7.7)
**CSF biomarkers**	
Aβ_42_, pg/mL, mean (SD)	750 (258)
Below threshold of 700 pg/mL, n (%)	33 (51.6)
T-tau, pg/mL, mean, (SD)	523 (442)
Above age adjusted threshold, n (%)	29 (45.3)
P-tau, pg/mL, mean, (SD)	70 (41)
Above threshold, n (%)	20 (31.3)
**Stage and clinical diagnosis combined**	
MCI-AD, n (%)	7 (11)
MCI-non-AD, n (%)	10 (16)
Dementia-AD, n (%)	34 (53)
Dementia-non-AD, n (%)	8 (12)
SCD-non-AD, n (%)	5 (8)
**Cognitive measures**	
MMSE, mean (SD) ^a^	25.32 (3.87)
CDR, mean (SD) ^b^	3.47 (2.26)

n, number of patients; y, years; CSF, cerebrospinal fluid; Aβ_42_, amyloid β; T-tau, total tau; P-tau, phosphorylated tau; MCI, mild cognitive impairment; AD, Alzheimer´s disease in terms of clinical etiology; non-AD, clinical etiology other than Alzheimer´s disease; SCD, subjective cognitive decline; MMSE, Mini-mental state examination; CDR, Clinical dementia rating scale.

^a^ n = 63, one patient did not have available MMSE

^b^ n = 63, one patient had no information to score CDR

### Flut+ and Flut- group differences

**Fig 1:**
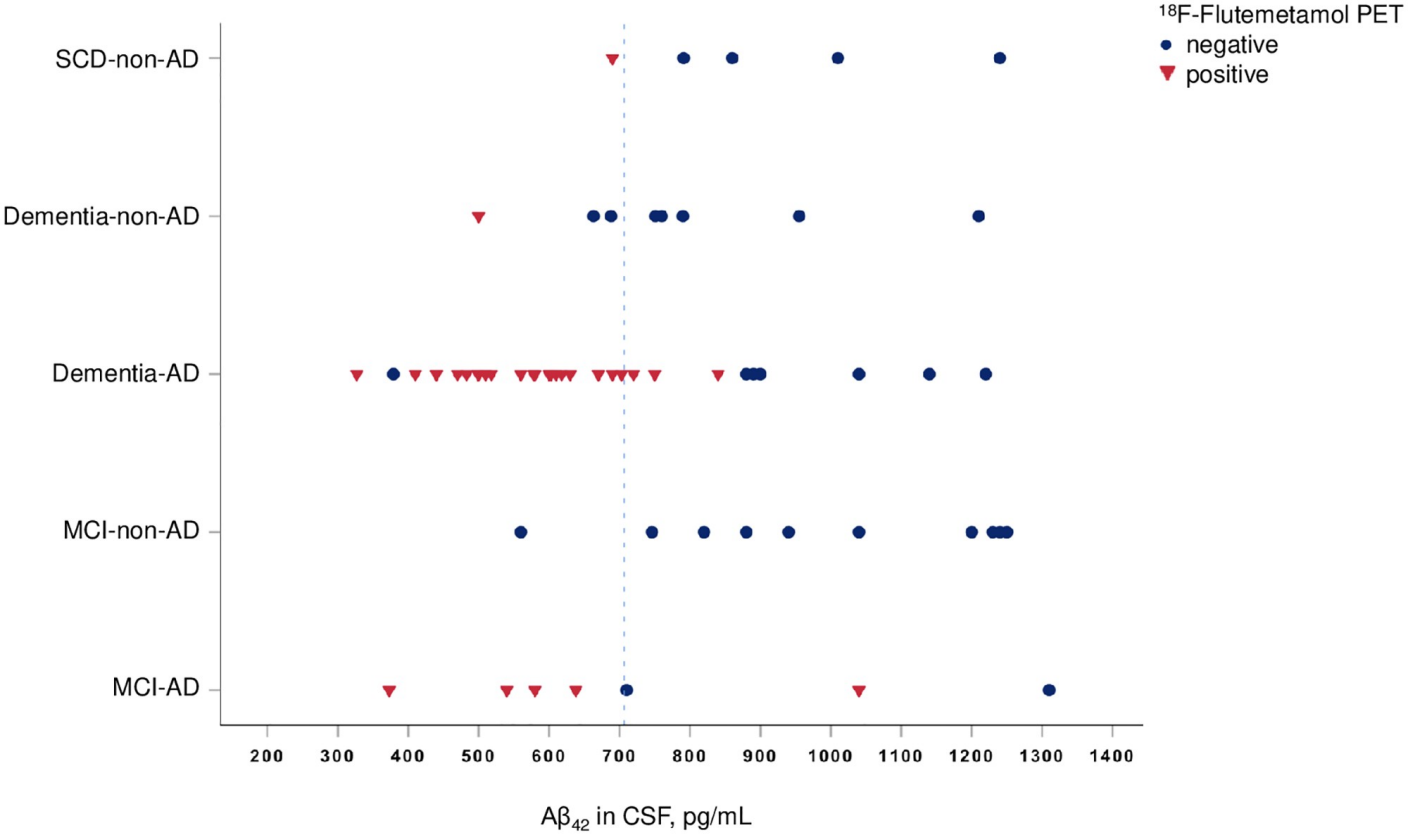
Graphic presentation of the combination of clinical diagnosis, Aβ_42_ value and ^18^F-Flutemetamol PET classification in all study participants. SCD, Subjective cognitive decline; AD, Alzheimer´s disease; MCI, mild cognitive impairment; PET, Positron emission tomography; Aβ_42_, amyloid β; CSF, cerebrospinal fluid. The blue dotted line represents the optimal cut-off value for Aβ_42_ derived from the ROC curve analysis presented (706.5 pg/mL).

Thirty-four ^18^F-Flutemetamol PET images (53%) were classified Flut+ and 30 images (47%) were classified Flut-. Differences in CSF biomarkers and ratios, diagnoses and cognitive measures in the Flut+ and Flut- groups are listed in Table 2 and Fig 1.

**Table 2:** Patient characteristics in ^18^F-Flutemetamol PET positive and negative groups.

	^18^F-Flutemetamol PET positive	^18^F-Flutemetamol PET negative	Group difference, *p* value
**Sex and age**			
Females, n	22	10	**0.012** ^a^
Males, n	12	20	
Total number of patients, n	34	30	-
Age, y, mean (SD)	68.4 (6.6)	63.9 (8.0)	**0.016** ^b^
**CSF biomarkers**			
Aβ_42_, pg/mL, mean (SD)	585 (137)	936 (236)	**<0.0001** ^c^
T-tau, pg/mL, mean, (SD)	635 (488)	396 (349)	**<0.001** ^c^
P-tau, pg/mL, mean, (SD)	85 (47)	54 (24)	**<0.002** ^c^
T-tau/Aβ_42_ ratio (SD)	1.2269 (1.43)	0.4485 (0.42)	**<0.0001** ^c^
P-tau/Aβ_42_ ratio (SD)	0.1574 (0.123)	0.0621 (0.042)	**<0.0001** ^c^
**Stage and clinical diagnosis combined**			
MCI-AD, n	5	2	-
MCI-non-AD, n	0	10	-
Dementia-AD, n	27	7	-
Dementia-non-AD, n	1	7	-
SCD-non-AD, n	1	4	-
**Clinical diagnosis**			
AD, n	32	9	**<0.0001** ^a^
Non-AD, n	2	21	
**Cognitive measures**			
MMSE, mean (SD)	24.53 (4.21)	26.24 (3.26) ^d^	0.08 ^b^
CDR, mean (SD)	3.75 (2.12)	3.14 (2.41) ^e^	0.29 ^b^

n, number of patients; y, years; CSF, cerebrospinal fluid; Aβ_42_, amyloid β; T-tau, total tau; P-tau, phosphorylated tau; MCI, mild cognitive impairment; AD, clinical diagnosis of Alzheimer´s disease; non-AD, clinical diagnosis other than Alzheimer´s disease; SCD, subjective cognitive decline; MMSE, Mini-mental state examination; CDR, Clinical dementia rating scale.

^a^ Pearson Chi-Square test

^b^ Independent samples T-test

^c^ Mann-Whitney *U* test

^d^ n = 29, one patient did not have available MMSE

^e^ n = 29, one patient had no information to score CDR

### Correlation of biomarkers and cut-off values

The univariate logistic regression analyses showed that all three biomarkers in CSF (Aβ_42_: *p* <0.0001, P-tau: *p* = 0.006, T-tau: *p* = 0.0047) as well as age (*p* = 0.021) and sex (*p* = 0.014) had a significant correlation to ^18^F-Flutemetamol PET. The odds-ratios presented below account for a 1 unit increase in each of the independent variables. The univariate analysis shows that the likelihood of a positive ^18^F-Flutemetamol PET is lower with increasing Aβ_42_ (odds ratio 0.366 for a 100 unit increase) and higher with increasing P-tau (odds ratio 2.499 for a 30 unit increase), T-tau (odds ratio 1.221 for a 100 unit increase), age and female gender. See Table 3 for summary.

**Table 3:** Univariate and multivariate logistic regression analyses for correlation with ^18^F-Flutemetamol positron emission tomography.

	Odds ratio ^a^	*p* value	95% confidence interval for odds ratio	Explained variance, R^2^
**Univariate logistic regression analysis**				
CSF Aβ_42_	0.990	**<0.0001**	0.985–0.995	0.608
CSF P-tau	1.031	**0.006**	1.008–1.053	0.220
CSF T-tau	1.002	**0.047**	1.000–1.004	0.123
Age	1.089	**0.021**	1.013–1.170	0.118
Sex ^b^	0.273	**0.014**	0.097–0.768	0.127
**Multivariate logistic regression analysis**				
CSF Aβ_42_	0.990	**0.001**	0.984–0.996	0.743
CSF P-tau	1.073	0.091	0.989–1.166	
CSF T-tau	0.996	0.450	0.987–1.006	
Age	1.186	**0.029**	1.018–1.383	
Sex ^b^	0.176	0.071	0.027–1.161	

CSF, cerebrospinal fluid; Aβ_42_, amyloid β; T-tau, total tau; P-tau, phosphorylated tau

^a^ Odds ratios account for a 1 unit increase in the respective variables.

^b^ Female is coded as 1 and male is coded as 2, odds ratio is given relative to 1

**Fig 2:**
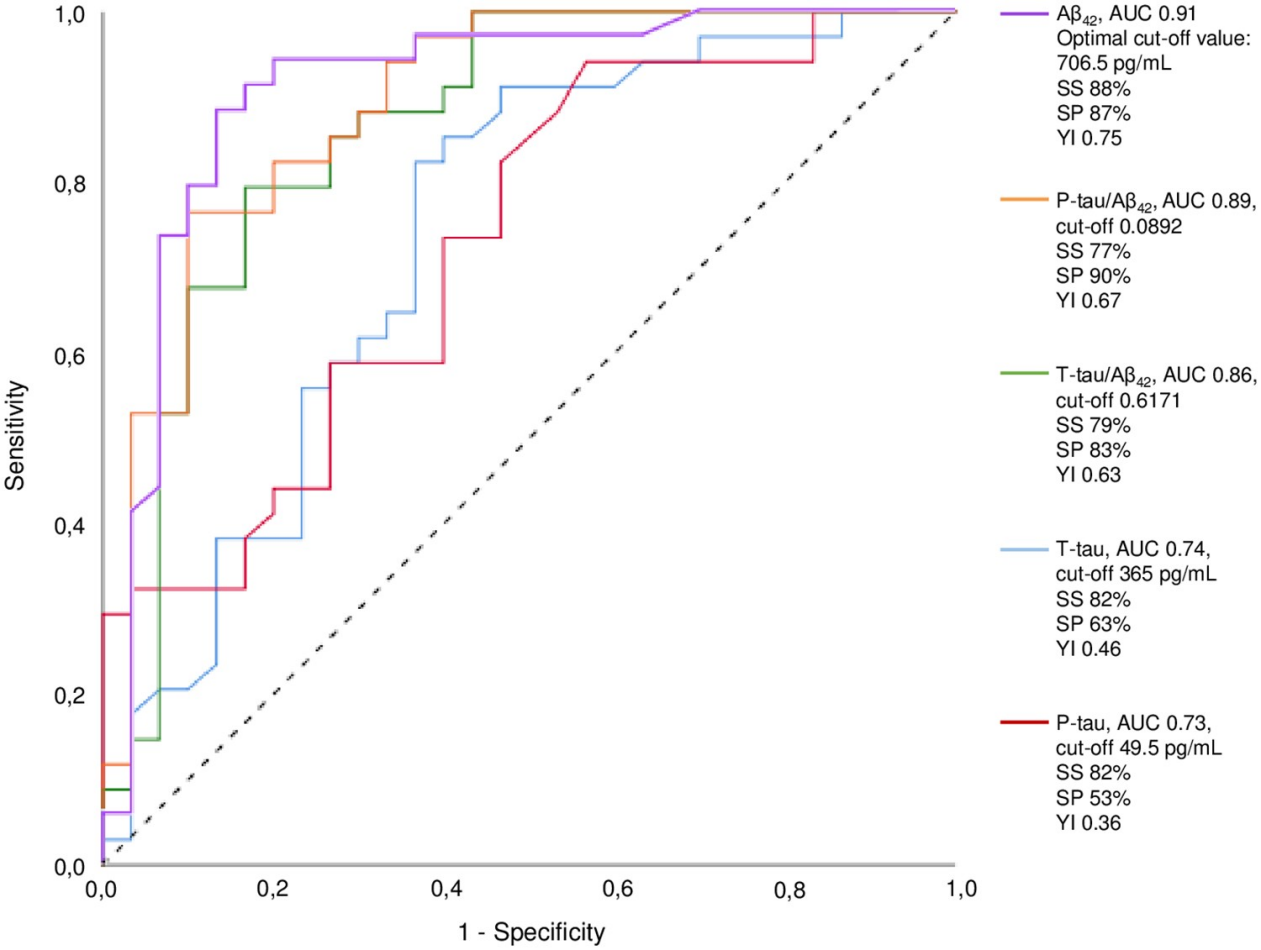
ROC curves for CSF biomarkers and ratios with ^18^F-Flutemetamol PET as classifier. The optimal cut-off value corresponding to the highest Youden´s index is given in the figure text. ROC, Receiver operating characteristic; CSF, Cerebrospinal fluid; PET, positron emission tomography; Aβ_42,_ amyloid beta; T-tau, total tau; P-tau, phosphorylated tau; AUC, area under the curve; SS, sensitivity; SP, specificity; YI, Youden´s index.

Aβ_42_ (*p* < 0.0001) and P-tau (*p* = 0.006) demonstrated the strongest correlation with ^18^F-Flutemetamol PET. The multivariate regression analysis showed that Aβ_42_ (*p* = 0.001) and age (*p* = 0.029) were the only significant predictors in the selected prediction model. The ROC curve for Aβ_42_ yielded an area under the curve (AUC) of 0.91. The highest Youden´s index (0.75) applied to an Aβ_42_ cut-off of 706.5 pg/mL (sensitivity 88% and specificity 87%). When applying the old cut-off value of 550 pg/mL this yielded a sensitivity of 41% and specificity of 97%, corresponding to a Youden´s index of 0.38. Summary of results from the ROC curves for CSF biomarkers and ratios are shown in Fig 2.

### Predicting clinical diagnosis

Thirty-two of the 34 patients (94%) with a positive ^18^F-Flutemetamol PET and nine of the 30 patients (30%) with a negative ^18^F-Flutemetamol PET had a clinical AD diagnosis. Results of the logistic regression analysis for predicting the likelihood for clinical AD diagnosis are listed in Table 4.

**Table 4:** Univariate and multivariate logistic regression analyses for prediction of clinical diagnosis.

	Odds ratio ^a^	*p* value	95% confidence interval for Odds ratio	Explained variance, R^2^
**Univariate logistic regression analysis**				
^18^F-Flutemetamol PET result ^b^	37.333	**<0.0001**	7.329–190.172	0.54
CSF Aβ_42_ ^c^	0.996	**0.001**	0.993–0.998	0.26
Age	1.107	**0.008**	1.027–1.194	0.16
Sex ^d^	0.280	0.218	0.094–0.831	0.12
CDR	1.123	0.126	0.944–1.600	0.06
**Multivariate logistic regression analysis**				
^18^F-Flutemetamol PET result ^b^	30.358	**0.002**	3.642–253.074	0.59
CSF Aβ_42_ ^c^	1.001	0.70	0.997–1.004	
Age	1.076	0.151	0.974–1.189	
Sex ^d^	0.395	0.240	0.084–1.859	
CDR	1.160	0.364	0.842–1.599	

PET, Positron emission tomography; CSF, cerebrospinal fluid; Aβ_42_, amyloid β; CDR, Clinical dementia rating scale.

^a^ Odds ratios account for a 1 unit increase in the respective variables.

^b^ Negative PET is coded as 0 and positive PET is coded as 1, odds ratio is given relative to 0

^c^ Measured on a continuous scale

^d^ Female is coded as 1 and male is coded as 2, odds ratio is given relative to 1

The univariate analysis showed that ^18^F-Flutemetamol PET (*p* < 0.0001), Aβ_42_ (*p* = 0.001) and age (*p* = 0.008) were significant, however, sex and CDR were not significant predictors of diagnosis. The odds ratios presented in Table 4 account for a 1 unit increase for each of the independent variables. The univariate analysis show that the likelihood of a clinical AD diagnosis was lower with increasing Aβ_42_ (odds-ratio 0.670 for a 100 unit increase) and higher with increasing age (2.763 with a 10 unit increase). Multiple models with different combinations of independent variables were tested in the multivariate analysis, among them also models including all CSF biomarkers together with ^18^F-Flutemetamol PET. ^18^F-Flutemetamol PET remained the most significant predictor in all the tested models. In the multivariate analysis of the selected prediction model, we found that ^18^F-Flutemetamol PET (*p* < 0.0001) was the only significant predictor, probably also due to collinearity of several parameters. The explained variance (R^2^) of this model was just slightly higher (0.59), compared to the R^2^ in the univariate analysis (0.54) for ^18^F-Flutemetamol PET on clinical diagnosis.

### Interrater agreement

Cohens κ showed a very good agreement between the readers classification of images (κ = 0.969, 95% CI: 0.91–1.03, *p* < 0.001) and in clinical diagnoses with and without knowledge of the ^18^F-Flutemetamol PET classification (κ = 0.953, 95% CI: 0.89–1.02, *p* < 0.001).

## Discussion

In the present study we found a significant correlation between ^18^F-Flutemetamol PET classification and the three CSF biomarkers explored, with the highest correlation between Aβ_42_ and ^18^F-Flutemetamol PET. The optimal cut-off value for Aβ_42_ presented is in accordance with the newly implemented cut-off value and yielded an improvement in sensitivity, while maintaining a high specificity, for a positive ^18^F-Flutemetamol PET. ^18^F-Flutemetamol PET was found to be the best predictor of a clinical AD diagnosis.

The significant correlation between the three CSF biomarkers and ^18^F-Flutemetamol PET is in line with previous studies [18–20]. The laboratory analyzing CSF samples for AD biomarkers in Norway recently elevated the recommended cut-off value for Aβ_42_ from 550 pg/mL to 700 pg/mL [23]. Our results support this change for use in Norwegian memory clinic settings. The change in cut-off value has recently been showed to also improve the sensitivity with only a small decrease in specificity for a clinical AD diagnosis and without increasing false positives [35]. A previous study compared different immunoassays with visual ^18^F-Flutemetamol PET and when using Innotest they found a cut-off value of 548 pg/mL (sensitivity 96% and specificity 82%) [36]. Other studies comparing ^18^F-Flutemetamol PET and Innotest for CSF analysis found a cut-off value of 647 pg/mL (sensitivity 95% and specificity 90%) [18], while two other studies found a cut-off value of 679 pg/mL (sensitivity 100% and specificity 89%) [37] and a cut-off value range of 645–762 pg/mL across different brain regions (sensitivities 87–93% and specificities 85–93%) [23]. For the other biomarkers we found that T-tau and P-tau had lower specificity for ^18^F-Flutemetamol PET than Aβ_42_, not surprisingly, as these are biomarkers of pathological processes that are not directly evaluated with ^18^F-Flutemetamol PET. The ratios performed better, understandingly, as they include Aβ_42_. Our cut-off values for T-tau, P-tau and the ratios were within the range found in other studies [19, 20, 36, 38, 39], although they are not directly comparable due to differences in patient populations, diagnostic criteria, CSF analysis methods as well as PET tracers and classification methods used. These factors represent a challenge when comparing studies exploring PET and CSF biomarkers and may partly also explain the differences in cut-offs for Aβ_42_ shown across studies. We did not have available Aβ_40_ in CSF. This would have been favorable to explore as the Aβ_42_/Aβ_40_ ratio is found to have better diagnostic accuracy as well as being a better predictor of abnormal Aβ PET than Aβ_42_ alone [40, 41].

We found that ^18^F-Flutemetamol-PET is a better predictor of a clinical AD diagnosis than Aβ_42_, however we suggest they can be used interchangeably for evaluating Aβ load in a memory clinic setting, due to the high correlation between ^18^F-Flutemetamol PET and Aβ_42_. This high correlation may have influenced the significance level of Aβ_42_ in the multivariate analysis, however ^18^F-Flutemetamol PET demonstrated a lower *p*-value as well as a higher explained variance than Aβ_42_ in the univariate analyses. ^18^F-Flutemetamol was also the most significant variable in all the tested prediction models. Which biomarker examination is used in clinical practice will vary depending on availability, tradition, patient comfort as well as confidence in performing and contraindications (e.g anticoagulative medications) for a lumbar puncture. However, Aβ_42_ level in CSF and Aβ PET does not provide identical information. The level of Aβ_42_ provides information about the Aβ_42_ epitopes that may be in either soluble or protofibrillar forms in CSF and reflects the balance between Aβ_42_ production and clearance at the time of lumbar puncture. It is further an indirect measure which is associated with the accumulation of Aβ in the form of plaques [6]. ^18^F-Flutemetamol-PET, on the other hand, provides information of the amount of insoluble Aβ fibrils and consequently the Aβ burden in the brain which has accumulated over time [42]. Previous longitudinal studies have described a nonlinear correlation between Aβ PET imaging and CSF Aβ_42_ which supports the hypothesis that the two methods show different aspects of Aβ pathology [43–45]. In the present study, there were eight patients (12.5%) with discordance between Aβ PET and CSF Aβ_42_ (using cut-off of 706.5 pg/mL), in seven of these there was concordance between PET and clinical diagnosis which explains the better performance of ^18^F-Flutemetamol PET as a predictor of clinical AD diagnosis (see Fig 1).

There were 11 patients (15%) with discordance between Aβ PET and clinical diagnosis (see Fig 1). All except one had concordance between Aβ PET and CSF Aβ_42_ (using cut-off of 706.5 pg/mL), in which eight had no evidence of Aβ pathology combined with a clinical AD diagnosis and two had evidence of Aβ pathology combined with a clinical non-AD diagnosis. One review reported a proportion of 2–32% of patients with a clinical AD diagnosis with a negative Aβ PET [46], while others have found this proportion to be as high as 61.3% [47]. The NIA-AA criteria applied here focuses on the clinical presentation where biomarkers are to be used only for support to refine confidence in the diagnosis. Although, the majority of patients without evidence of Aβ pathology (either PET or CSF) and clinical AD diagnosis (both dementia and MCI) in this study did have evidence of neurodegeneration. These cases may be categorized as “Suspected non-Alzheimer disease pathophysiology” (SNAP), which is a biomarker-based concept in which evidence of Aβ deposition is lacking, but evidence of neurodegeneration is present [48]. The sensitivity of the NIA-AA criteria has previously been reported with sensitivities ranging from 65.6% to 79.5% for probable and possible AD respectively, but with specificities considerably higher [49]. Whether biomarkers are to be included in the clinical criteria to further improve their sensitivity is still under investigation.

Another explanation for a negative Aβ PET with a clinical AD diagnosis is the possibility of a false-negative PET. This is thought to occur if non-fibrillar, smaller or less dense Aβ species are dominating, which may be challenging to detect by qualitative Aβ PET [50]. We did not apply quantification methods to evaluate ^18^F-Flutemetamol PET as this study reflects clinical practice in which qualitative classification is used, which is currently the only validated method [14, 21]. Although one autopsy study found that ^18^F-Flutemetamol can detect also diffuse Aβ plaques [16, 51], the ability of Aβ PET to detect the smallest and oligomeric toxic Aβ species is unlikely. Wolk *et al* included both qualitative and quantitative classification of images and found similar results using the two methods [52]. In preclinical stages of the AD continuum however, it is possible that quantitative PET will provide an even higher sensitivity for Aβ which may be of value, especially in drug trials. Cortical Aβ burden demonstrated with PET is nevertheless a sensitive biomarker, without being specific, for AD and its effect on diagnoses and outcomes is still under investigation. This stress the importance of an appropriate use of Aβ PET imaging [53].

The women included in this study had more evidence of Aβ pathology. There were no significant differences in age, MMSE, CDR, T-tau or P-tau between females and males. However, a larger proportion of women also had a clinical AD diagnosis. Whether this indicate more severe disease at the time of diagnostic workup in women remains unclear. Sex differences have been reported previously [54], the occurrence of AD and other forms of dementia has been shown to be higher in women [2, 55] and studies show that in cognitively normal individuals men are more likely to have SNAP than women [48]. In the presented population we found eight patients (12,5%) that may have SNAP and five of these were males.

The demonstrated excellent interrater agreement of ^18^F-Flutemetamol PET in this study have been reported to be similarly high in other studies [21, 52], which confirms that the qualitative method is a highly robust and effective way to classify Aβ PET in patients in a memory clinic setting. Despite one of the memory clinic physicians who made diagnoses were blind to the ^18^F-Flutemetamol PET result, the interrater agreement of diagnoses was excellent. However, both physicians had the Aβ_42_ level in CSF available, suggesting that these methods for evaluating Aβ load may be used interchangeably to support a clinical diagnosis.

A limitation of this study is the rather low number of patients included, which is partly a consequence of excluding patients with more than 190 days between PET and lumbar puncture. All data was collected from clinical practice which may be regarded as both a strength and a limitation. The inclusion was performed through a memory clinic with a preselected population with typically younger patients with most likely more challenging diagnoses. We did not include information about the *APOE* genotype nor did we have a control group, both which would have strengthened the study. We did not perform semi-quantification of ^18^F-Flutemetamol PET, mainly due to lack of an appropriate MRI for such purposes. Semi-quantification could have provided an objective evaluation of amyloid burden as well as improved the correlations to CSF biomarkers further and is consequently a limitation of this study. All CSF analyses were performed according to the same routine, analyzed in the same laboratory and all PET images were acquired at the same PET scanner with the same standardized protocol, which are considered strengths of this study.

In the present study we found a high correlation between qualitative ^18^F-Flutemetamol PET and Aβ_42_ in CSF in patients referred from a memory clinic and ^18^F-Flutemetamol PET was the best predictor of clinical AD diagnosis. Thus, ^18^F-Flutemetamol PET is a valuable tool to refine confidence that a patients’ cognitive impairment is caused by pathophysiological changes in the AD continuum.
